# The Mitochondrial Genome of *Curcuma longa*: A Large and Structurally Complex Genome with Extensive Intracellular DNA Transfer

**DOI:** 10.3390/genes17020243

**Published:** 2026-02-19

**Authors:** Bing Xu, Minlong Jia, Jiali Kong, Liyun Nie, Jie Wang, Luke R. Tembrock, Zhiqiang Wu, Sen Li, Xuezhu Liao

**Affiliations:** 1 College of Horticulture, Shanxi Agricultural University, Jinzhong 030801, China; xubing328@126.com (B.X.);; 2 Shenzhen Branch, Guangdong Laboratory of Lingnan Modern Agriculture, Key Laboratory of Synthetic Biology, Ministry of Agriculture and Rural Affairs, Agricultural Genomics Institute at Shenzhen, Chinese Academy of Agricultural Sciences, Shenzhen, 518124, China; 3 Department of Agricultural Biology, Colorado State University, Fort Collins, CO 80523, USA; 4 Guangdong Provincial Key Laboratory for Plant Epigenetics, College of Life Sciences and Oceanography, Shenzhen University, Shenzhen 518060, China

**Keywords:** plant mitochondrial genome, organelle structure, genome expansion, NUMTs, NUPTs

## Abstract

**Background**: Plant mitochondrial genomes exhibit extreme variation in size and structure while maintaining a conserved set of core protein-coding genes. This combination of structural diversity and functional conservation provides valuable insights into evolutionary processes such as genome expansion, rearrangement, and intracellular DNA transfer. *Curcuma longa*, an economically and medicinally important species in the genus *Curcuma* (Zingiberaceae), has not yet been studied in terms of the organization and evolution of its mitochondrial genome. **Methods**: In this study, we assembled and annotated the mitochondrial and plastid genomes of *C. longa* using third-generation HiFi sequencing data, systematically analyzing their genomic structure, repetitive sequence content, and features of sequence transfer between nuclear and organellar genomes. **Results**: The mitochondrial genome of *C. longa* was assembled as a complex, network-like structure consisting of 12 contigs with a total length of approximately 7.7 Mb, making it one of the largest mitochondrial genomes reported in monocots to date. Comparative analysis revealed significant differences in repeat types, abundance, and length distribution between the two organellar genomes. Additionally, extensive intracellular DNA transfer events were identified among the nuclear, mitochondrial, and plastid genomes. **Conclusions**: Overall, this study provides the first comprehensive report on the giant mitochondrial genome of *C. longa*, detailing its structural organization, repeat content, and intergenomic transfers. These findings lay a foundation for understanding mitochondrial genome evolution in *Curcuma* and offer broader insights into the mechanisms driving extreme mitochondrial genome expansion in angiosperms and monocots specifically.

## 1. Introduction

The nuclear, mitochondrial, and plastid genomes collectively govern cellular function and evolution in plants. Among these, mitochondrial genomes exhibit extraordinary diversity in size and structural organization across land plants [1,2,3,4]. In contrast to plastid genomes, which are compact and structurally conserved, plant mitochondrial genomes display extensive rearrangements, multipartite and reticulate architectures, and dramatic genome expansion [5]. Despite this structural complexity, the repertoire of protein-coding genes remains highly conserved. Unlike animal mitochondrial genomes, plant mitogenomes are characterized by dynamic structural variations within individuals and often exist as a heterogeneous population of alternative isoforms rather than a single master circle [6]. This dynamic nature involves frequent homologous recombination mediated by repetitive sequences, which can generate sub-stoichiometric molecules and facilitate rapid genomic rearrangement [7,8]. Understanding these mechanisms, specifically how recombination and repeat accumulation drive structural divergence and heteroplasmy, remains a central challenge in plant genomics [9]. However, recent advances in sequencing technologies [10,11,12], and the development of dedicated assembly tools [13,14,15,16,17,18], have provided the necessary resolution to decipher these complex architectures, thereby supporting deeper investigation into organellar biology. Nevertheless, mitochondrial genome research lags behind that of plastids in both taxonomic breadth and structural diversity [3,19]. Most available plant mitochondrial genomes are from species with small and simple genomes, whereas those with exceptionally large mitochondrial genomes remain underrepresented [20,21]. This sampling bias limits our understanding of the mechanisms driving mitochondrial genome expansion and structural diversification.

A distinctive feature of plant mitochondrial genomes is their extreme size variation, which spans more than two orders of magnitude from 66 kb in *Viscum scurruloideum* [22] to 18.9 Mb in *Cathaya argyrophylla* [23]. This expansion is largely attributed to the accumulation of non-coding DNA, recombination mediated by repetitive sequences, and the uptake of foreign DNA from plastid or viral sources [24,25]. However, the limited number of available large mitochondrial genomes hinders comparative analysis of repeat content, recombination patterns, and sequence transfer, leaving key questions about their formation and evolutionary constraints unanswered. This is particularly true for the Zingiberaceae family, a group known for its ecological and economic significance. *C*. *longa*, an economically important species in the Zingiberaceae family, is widely utilized for its distinctive curcuminoid compounds present in the rhizomes for medicine, food, and cosmetics [26,27]. While plastid genomes of over 20 *Curcuma* species have been reported and used in phylogenetic studies [28], mitochondrial genomes in this group remain largely uncharacterized. To date, only one mitochondrial genome, that of *Curcuma amarissima*, has been published, which was assembled into 39 contigs with a multi-branched structure [29], suggesting a highly complex and potentially expanded genome architecture. Therefore, resolving the true structural configuration of such complex genomes is essential for understanding the evolutionary forces driving mitogenome expansion in Zingiberaceae.

In this study, we assembled the complete mitochondrial and plastid genomes of *C. longa* using PacBio HiFi sequencing. Through comparative analysis with the fragmented *C. amarissima* genome, we aimed to: (1) resolve the highly complex, reticulate architecture of the *C. longa* mitogenome and clarify specific structural variations; (2) systematically identify repetitive sequences, including simple sequence repeats (SSRs), tandem repeats (TRs), and dispersed repeats, enabling a quantitative evaluation of their contribution to recombination-mediated mitochondrial genome expansion; (3) characterize intracellular DNA transfers, such as NUMTs (nuclear mitochondrial DNA segments), NUPTs (nuclear plastid DNA segments), and MTPTs (plastid-derived integrations into the mitochondrial genome), detailing their length, distribution, and insertion sites. These analyses allowed us to assess the evolutionary consequences of sequence transfer on mitochondrial genome organization. This work provides a high-quality genomic reference for *Curcuma* and advances our understanding of the mechanisms driving mitochondrial genome enlargement in Zingiberaceae.

## 2. Materials and Methods

### 2.1. Data Source

*C. longa* (Zingiberaceae) plants were cultivated in a greenhouse of the Institute of Agricultural Genomics at Shenzhen, Chinese Academy of Agricultural Sciences. Plants were grown under natural photoperiod conditions with temperatures maintained at approximately 26–28 °C. Young, healthy leaves were collected from mature plants for DNA extraction and PacBio HiFi sequencing. The whole-genome HiFi sequencing data of *C. longa* were generated previously by our research group [30] and were used for subsequent organellar genome assembly in the current study.

### 2.2. Organellar Genome Assembly and Annotation

We extracted 4 million reads from the whole-genome HiFi dataset of *C. longa* and used them as input for PMAT v1.5.3 [16] to enrich organellar reads and generate preliminary assemblies. To refine the mitochondrial assembly, mitochondrial sequences from this step served as bait to identify all mitochondrial reads from the full dataset via BLAST v2.15.0+ [31]. Approximately 300,000 randomly selected mitochondrial reads were then reassembled using PMAT v1.5.3 [16]. The assembly graph was visualized and simplified in Bandage v0.8.1 [32] to produce the final mitochondrial contigs. Plastid assembly followed an identical strategy. Assembly completeness and coverage were assessed by mapping final contigs to the original HiFi reads using minimap2 v2.1 [33] and inspecting the alignments in IGV v2.16.0 [34].

Mitochondrial genome annotation was performed with PMGA [35] for protein-coding and rRNA genes and tRNAscan-SE v2.0 [36] for tRNA genes. To assist annotation accuracy, alignments with monocot mitochondrial reference genes were performed using MAFFT v7.490 [37] and BioEdit v7.7.1 [38]. Gene boundaries were subsequently manually verified and corrected in Apollo 1.11.8 [39]. Plastid genome annotation was performed using GeSeq [40], with the *Curcuma alismatifolia* reference genome, and was subsequently manually validated in Apollo 1.11.8 [39].

### 2.3. Phylogenetic Reconstruction and Evolutionary Analysis

For 13 monocotyledonous species, protein-coding genes (PCGs) shared across the mitochondrial genomes and a separate set shared across the plastid genomes were selected. Specifically, 12 shared mitochondrial genes and 42 single-copy plastid genes were identified. The species analyzed included *Aechmea fasciata* (NC_087835.1), *Avena longiglumis* (NC_072961.1), *Cocos nucifera* (NC_031696.1), *C. amarissima* (PQ442956.1-PQ442994.1), *Oryza sativa* (NC_007886.1), *Phoenix dactylifera* (NC_016740.1), *Poa pratensis* (NC_077587.1), *Pontederia crassipes* (NC_084342.1), *Sorghum bicolor* (NC_008360.1), *Zantedeschia aethiopica* (NC_073008.1), *Zea mays* (NC_007982.1), *Saccharum officinarum* (NC_031164), and *Dracaena cochinchinensis* (NC_086589.1), with *C. nucifera* set as the outgroup. Gene sequences were aligned using MAFFT v7.490 [37], trimmed with TrimAl v1.4.rev15 [41], and concatenated into super matrices with SeqKit2 [42]. Maximum likelihood phylogenies were inferred using IQ-TREE v3.0.1 [43] with 1000 ultrafast bootstrap replicates.

To assess mitochondrial genome size variation, annotated angiosperm assemblies were retrieved from the NCBI organelle genome database (https://www.ncbi.nlm.nih.gov/datasets/organelle/, (accessed on 15 December 2025)). The largest (18.99 Mb) and smallest (66 kb) assemblies were excluded to minimize outlier bias. The size distribution of the filtered dataset was visualized as density plots using R v4.5.2.

Synteny between the mitochondrial genomes of *C. longa* and *C. amarissima* was identified using BLASTN v2.15.0+ [31]. Alignments with >80% sequence identity and length > 500 bp were retained and visualized as a Circos-style plot generated using the R package circlize v0.4.15 [44].

### 2.4. Codon Usage in the C. longa Mitochondrial Genome

Protein-coding gene (PCG) sequences were manually extracted from the mitochondrial genome. Only genes longer than 300 bp that possessed standard initiation codons and canonical termination codons were retained for subsequent analyses (Table S2).

Codon usage patterns and nucleotide composition were analyzed using CodonW v1.4.4 [45] with default parameters. This analysis was used to calculate GC content, relative synonymous codon usage (RSCU), and the effective number of codons (ENC). An ENC-GC3 plot was generated using the R package ggplot2 v4.0.0 [46]. The expected ENC curve was calculated following previously described methods using the formula:

$$\mathrm{ENC}\;=\;2\;+\;\mathrm{GC}3\;+\;\frac{29}{\left({\mathrm{GC}3}^{2}+\;{\left(1\;-\;\mathrm{GC}3\right)}^{2}\right)}$$

In addition, GC3–GC12 correlation analysis was performed, and the corresponding scatter plots were visualized using ggplot2 v4.0.0 [46].

### 2.5. Repeat Sequences and Shared DNA Analysis

Dispersed repeats within the *C. longa* mitochondrial and plastid genomes were identified by self-alignment using BLAST v2.15.0+ [31] (word size 7; E-value ≤ 1 × 10^−5^). Repeat pairs with length ≥ 30 bp and sequence identity ≥ 80% were retained. In the mitochondrial genome, repeats were grouped by length into four classes: Class I (30–200 bp), Class II (200–500 bp), Class III (500–1000 bp), and Class IV (≥1000 bp). Simple sequence repeats (SSRs) were detected with MISA v2.1 [47] using minimum repeat thresholds of 10, 8, 5, 4, 3, and 3 for mono- to hexa-nucleotide motifs, respectively. Tandem repeats (TRs) were identified with Tandem Repeats Finder v4.09 [48] using default parameters. To avoid redundancy, shorter repeats fully embedded within longer units were removed before calculating total repeat number and cumulative length.

In the plastid genome, dispersed repeats were further categorized by orientation as forward, reverse, or palindromic repeats. SSR detection thresholds were set to 10, 8, 4, 4, 3, and 3 for mono- to hexa-nucleotide motifs.

### 2.6. Mitochondrial Genome-Derived DNA Fragments in C. longa Nuclear Genome

To detect organellar DNA transfers, we aligned the assembled *C. longa* nuclear genome against its mitochondrial and plastid genomes using BLASTN v2.15.0+ [31]. Nuclear genome data were obtained from previously published work by our research group [30]. In addition, the mitochondrial genome was aligned to the plastid genome. Alignments meeting the following criteria were retained: E-value ≤ 1 × 10^−5^, length ≥ 100 bp, sequence identity ≥ 80%, and word size = 7. Overlapping or adjacent alignment fragments for each candidate transfer event were merged to generate non-redundant sets of nuclear mitochondrial DNA segments (NUMTs), nuclear plastid DNA segments (NUPTs), and mitochondrial plastid DNA segments (MTPTs).

## 3. Results

### 3.1. Assembly and Structural Features of the C. longa Mitochondrial Genome

**Figure 1:**
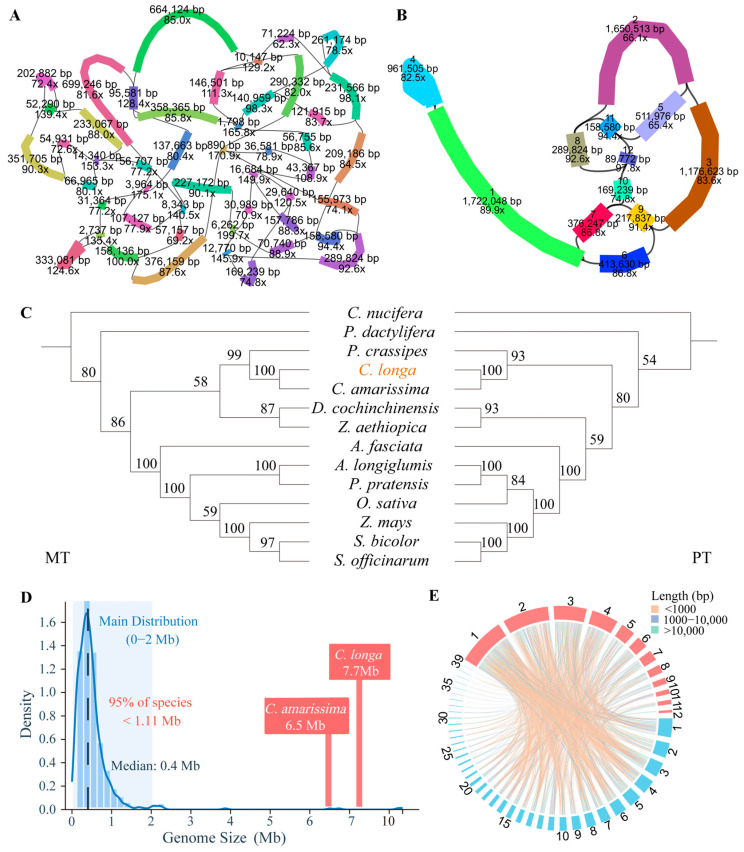
Assembly features and structural comparison of the *C. longa* mitochondrial genome: (**A**) Raw assembly graph of the *C. longa* mitochondrial genome; (**B**) Simplified assembly representation of the *C. longa* mitochondrial genome; (**C**) phylogenetic trees from mitochondrial data on the left and plastid data on the right, bootstrap values are shown at nodes; (**D**) Normal distribution of mitochondrial genome sizes based on publicly available plant mitochondrial genomes deposited in NCBI; (**E**) Circos-based synteny comparison between the mitochondrial genomes of *C. longa* (red) and *C. amarissima* (blue).

Using PacBio HiFi reads, we successfully resolved the mitochondrial genome of *C. longa*. The initial assembly of the *C. longa* mitochondrial genome displayed a highly complex network-like structure, yielding 48 contigs ranging from 890 bp to 699,246 bp, with a total assembly size of approximately 7.0 Mb (Figure 1A). By simplifying the graph topology and resolving collapsed repetitive sequences, the final assembly was resolved into 12 contigs with a total length of 7.7 Mb and a GC content of 43.9% (Figure 1B). Annotation identified 27 conserved protein-coding genes, 13 variable genes, 22 tRNA genes, and 3 rRNA genes (Table 1). Notably, despite the massive genome expansion, the gene content remains comparable to typical angiosperms, reflecting the evolutionary pattern where gene content is conserved even as genome size fluctuates. In sharp contrast to the expanded mitogenome, the plastid genome of *C. longa* assembled as a typical, compact angiosperm plastome of 162,180 bp, encoding 81 protein-coding genes (Figure S1; Table S1). The graphical visualization of the mitochondrial assembly revealed a highly complex, network-like architecture of the *C. longa* mitochondrial genome.

To contextualize the exceptional size of the *C. longa* mitogenome, we surveyed plant mitochondrial genome sizes using publicly available assemblies in the NCBI database, encompassing more than 600 species spanning over 350 genera and 100 families. This analysis showed that plant mitochondrial genome sizes span a broad range, from 66 kb to 18.9 Mb. The majority of reported genomes are concentrated within the 0–2 Mb interval, with most being under 1.11 Mb (Figure 1D). In this context, both *C. longa* (7.7 Mb) and its close relative *C. amarissima* (6.5 Mb) fall within the extreme upper tail of plant mitochondrial genome size distribution, representing exceptionally large mitochondrial genomes among angiosperms.

Despite the conserved gene content and massive size shared by *Curcuma* species, comparative analysis revealed striking structural divergence. We constructed trees based on shared protein-coding genes from both mitochondrial and plastid genomes, using 13 representative monocot species. In both the mitochondrial and plastid phylogenies, *C. longa* and *C. amarissima* formed a distinct clade, consistent with their taxonomic proximity (Figure 1C). However, this close evolutionary relationship is not mirrored in their genomic architecture. Synteny analysis revealed a complete breakdown of collinearity, with no syntenic blocks exceeding 50 kb (Figure 1E). Furthermore, approximately 25% of the *C. longa* sequence is species-specific and of unknown origin. This fragmented synteny reflects extensive structural divergence between the mitochondrial genomes of *C. longa* and *C. amarissima*.

### 3.2. Conserved Gene Content and Codon Usage in C. longa Mitogenome

**Figure 2:**
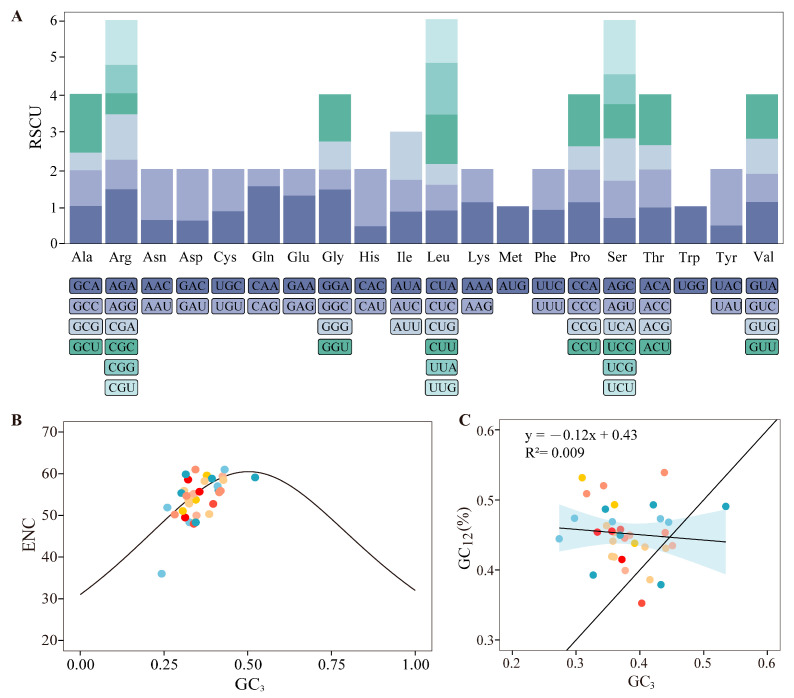
Codon usage and evolutionary constraints of protein-coding genes in the mitochondrial genome of *C. longa*: (**A**) Relative synonymous codon usage (RSCU) analysis; (**B**) Effective number of codons (ENC) scatter plot of protein-coding genes; (**C**) Neutrality plot analysis of protein-coding genes.

To characterize the coding gene content of the *C. longa* mitochondrial genome, we annotated all protein-coding and RNA genes. Annotation identified 40 protein-coding genes, along with 22 tRNA and 3 rRNA genes, a repertoire typical of angiosperms. To assess selective pressure on these coding regions, we analyzed codon usage patterns (Figure 2). The results indicated a weak overall codon bias, with effective number of codons (ENC) values ranging from 36 to 61 (Table S4). Over 90% of the genes exhibited ENC values exceeding 50, and notably low bias (ENC > 58) was detected in essential genes such as *rps14*, *matR*, and *nad9*. Consistent with other plant mitogenomes, a preference for A/T-ending codons was observed, but the RSCU values were generally balanced (0.46–1.57). Furthermore, no significant correlation was detected between GC12 and GC3. Protein-coding regions represent only a small proportion of the total 7.7 Mb mitochondrial genome assembly.

### 3.3. Repetitive Sequence Analysis in the C. longa Organellar Genomes

**Figure 3:**
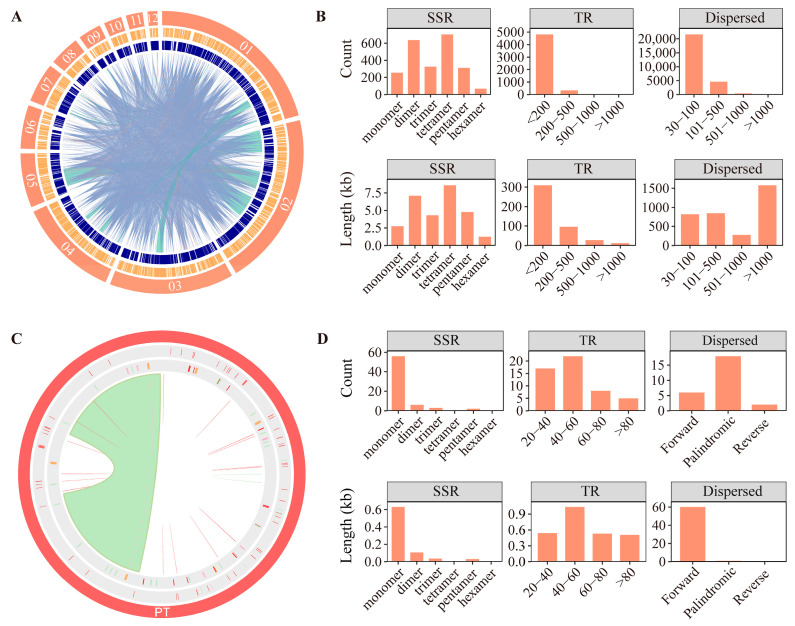
Repetitive sequences of mitochondrial and plastid genomes in *C. longa*: (**A**) Circos plot showing the distribution of repetitive sequences in the mitochondrial genome. The outermost circle represents mitochondrial chromosomes. The second outer circle indicates the distribution of SSRs, the next circle represents TRs, and the innermost circle shows dispersed repeats. Due to visualization constraints, only dispersed repeats longer than 300 bp are displayed; (**B**) Summary metrics of repetitive sequences in the mitochondrial genome; (**C**) Circos plot illustrating the distribution of repetitive sequences in the plastid genome; (**D**) Summary metrics of repetitive sequences in the plastid genome.

To elucidate the mechanisms underlying the pronounced genome expansion and structural rearrangement observed in the *C. longa* mitochondrial genome, we systematically characterized its repetitive sequence content, including SSRs, TRs, and dispersed repeats. In the mitochondrial genome, dispersed repeats were identified as the primary drivers of expansion. We detected 26,652 dispersed repeats, which covered 45.54% of the total genome length. Although ultra-long repeats (≥1000 bp) were infrequent (119 loci), they contributed disproportionately (44.8%) to the total length of dispersed repeats, providing abundant substrates for recombination. In addition to dispersed repeats, we identified 5296 TRs and 2302 SSRs, accounting for 5.74% and 0.37% of the genome, respectively. The SSRs were dominated by tetranucleotide motifs (~30%) and showed a uniform distribution across the genome (Figure 3A).

In sharp contrast to the mitochondrial genome, the plastid genome exhibited a much more compact architecture. Total repeats constituted 50% of the mitochondrial genome but only 38% of the plastid genome (Figure 3B,D). Specifically, the plastid genome contained far fewer repeats, with only 67 SSRs (0.49%) and 26 dispersed repeats detected. Notably, the majority of the repeat content in the plastid genome was attributed to the conserved large inverted repeat (IR) region (29,745 bp), rather than the pervasive proliferation of dispersed repeats observed in the mitochondrion.

### 3.4. Characterization of Transferred Fragments Between C. longa Genomic Compartments

**Figure 4:**
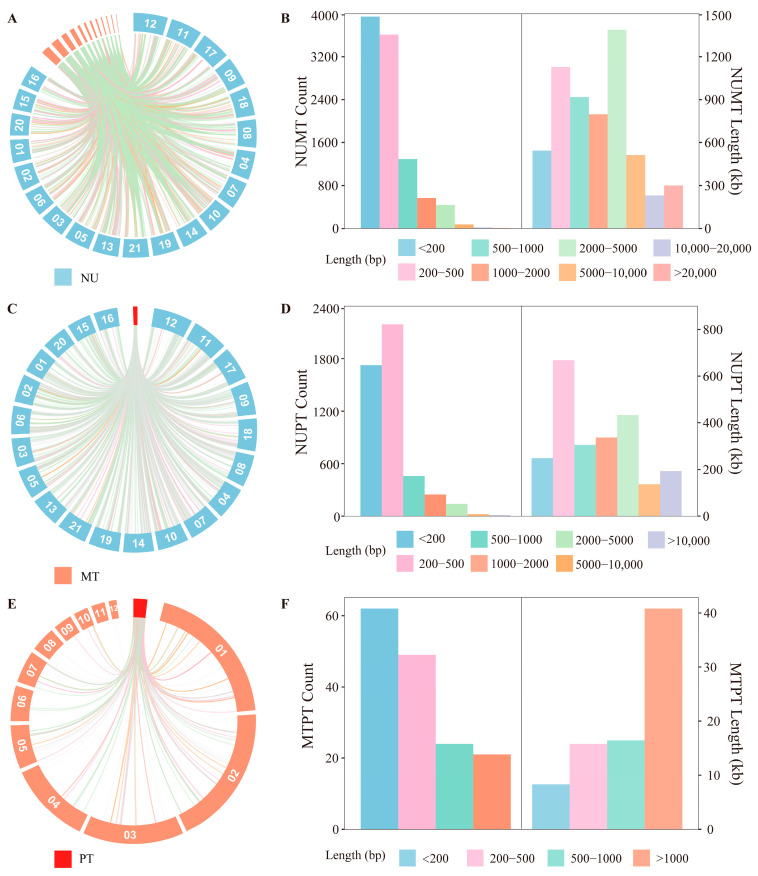
The characterization of inter-genomic DNA transfer. Chromosomes are ordered by descending length (from longest to shortest): (**A**) distribution of NUMT transfer fragments; (**B**) length and quantity distribution of NUMTs; (**C**) distribution of NUPT transfer fragments; (**D**) length and quantity distribution of NUPTs; (**E**) distribution of MTPT transfer fragments; (**F**) length and quantity distribution of MTPTs.

In addition to internal repeat-driven expansion, we further investigated whether intracellular DNA transfer among the nuclear, mitochondrial, and plastid genomes has contributed to the exceptional size of the *C. longa* mitochondrial genome. We identified 156 MTPTs integrated into the mitochondrial genome, totaling 81.37 kb. Representing only 1.06% of the total assembly, this relatively minor contribution indicates that intracellular transfer is not the primary driver of the observed gigantism. While short fragments (<500 bp) were most frequent, larger fragments (>1000 bp) contributed nearly half (48.5%) of the total MTPT length (Figure 4E,F). Furthermore, we systematically quantified mitochondrial and plastid-derived DNA fragments transferred into the nuclear genome. A total of 5.82 Mb of nuclear mitochondrial DNA segments (NUMTs) were identified (Figure 4A,B). The length distribution of NUMTs was highly skewed; while 83.5% were short (<500 bp), rare large fragments (>5000 bp) contributed 15.6% of the total transferred length. Similarly, 4790 nuclear plastid DNA segments (NUPTs) were detected, totaling 2.32 Mb (Figure 4C,D).

These results show that extensive intracellular DNA transfer occurs among the nuclear, mitochondrial, and plastid genomes of *C. longa*, with clear differences in both abundance and fragment length distribution among transfer categories. Among these, MTPTs account for only a small proportion of the total mitochondrial genome length.

## 4. Discussion

Extreme variation in genome size represents a defining feature of plant mitochondrial genome evolution [49,50,51,52]. While most angiosperm mitochondrial genomes are smaller than 1 Mb, advances in sequencing have led to the identification of numerous species possessing exceptionally large mitochondrial genomes, such as *Silene conica* (11.3 Mb), *Silene noctiflora* (6.7 Mb) [53] and *Larix sibirica* (11.6 Mb) [54]. In this study, we report a 7.7 Mb mitochondrial genome for *C. longa*, establishing it as one of the largest reported in monocots to date. Notably, this massive genomic expansion is not accompanied by any corresponding increase in coding capacity. The gene repertoire remains typical, consisting of only ~40 core genes. This extreme uncoupling between genome size and gene content reinforces the paradigm that plant mitogenomes evolve via a structural inflation–functional stasis mode. In this model, expansion is driven primarily by the accumulation of non-coding DNA and repetitive elements rather than functional innovation [55,56,57].

Repetitive sequences are considered major drivers of structural dynamism in plant mitochondrial genomes [58]. Dispersed repeats, often hundreds to thousands of base pairs in length, can serve as important loci for homologous recombination and mediate intra- or inter-molecular rearrangements [55,59,60], which are processes regulated by nuclear-encoded proteins such as RecA, OSB1, and MutS homologs [61]. In *C. longa*, dispersed repeats dominated the mitochondrial genome in both number and total length, accounting for approximately 45.54% of its sequence. Although long dispersed repeats (>1 kb) were few in number, they contributed substantially to total repeat length. This pattern agrees with observations from other structurally complex plant mitochondrial genomes [62]. It also suggests that recombination potential is largely influenced not by the abundance of short repeats, but by a limited set of long repeats capable of mediating frequent exchange [63,64]. In line with this, synteny analysis between *C. longa* and *C. amarissima* revealed highly fragmented overall homology and an absence of collinear blocks longer than 50 kb, despite approximately 75% sequence similarity. This combination of high sequence conservation and extreme structural divergence is characteristic of repeat-mediated recombination operating under low nucleotide substitution rates.

In addition to internal complexity, the high number of NUMTs detected in the *C. longa* nuclear genome reflects the dynamic nature of its mitochondrial DNA [65,66]. We identified 9954 NUMTs with a cumulative length exceeding 5.8 Mb, distributed in a pattern dominated numerically by short fragments but with substantial length contribution from long fragments. The abundance and length distribution of NUMTs in *C. longa* are therefore consistent with its large mitochondrial genome, rich repeat content, and fragmented structure, supporting the view that mitochondrial DNA undergoes recurrent breakage, recombination, and intracellular transfer [67,68]. Furthermore, the presence of NUPTs and MTPTs indicates ongoing genetic exchange among the three genomic compartments in *C. longa* [69,70], supplying additional repetitive templates that may facilitate further structural rearrangements [71].

In summary, the extraordinary expansion of the *C. longa* mitochondrial genome likely results from the combined action of multiple evolutionary processes. Accumulation of repetitive sequences, especially long dispersed repeats, provides a structural basis for frequent homologous recombination. Recurrent rearrangements continuously disrupt synteny and reshape genome architecture. Persistent DNA transfer among mitochondrial, plastid, and nuclear genomes introduces foreign sequences into the mitochondrial and nuclear compartments. Finally, a substantial proportion of sequences of unknown origin further contributes to the exceptionally large size and complexity of the *C. longa* mitochondrial genome [72].

## 5. Conclusions

Using PacBio HiFi sequencing, we assembled the mitochondrial and plastid genomes of *C*. *longa*. The mitochondrial genome is large (7.7 Mb) and structurally complex, with a branched architecture, multiple rearrangements, and abundant repeats. The plastid genome maintains a typical quadripartite structure.

Repeats differ substantially between the two genomes in type, abundance, and length distribution. Dispersed repeats, particularly those of substantial length, are markedly enriched in the mitochondrial genome and may drive its structural variation. In addition, we observed frequent transfer of mitochondrial DNA into the nuclear genome, whereas plastid-derived nuclear sequences were comparatively scarce. This pattern suggests an asymmetric model of intracellular DNA transfer from organelles to the nucleus.

Although *C. longa* and *C. amarissima* are closely related, their mitochondrial genomes show limited structural conservation, suggesting rapid divergence in genome architecture within the genus. Mitochondrial protein-coding genes display weak codon usage bias, consistent with low selection pressure on synonymous sites.

This study provides the first detailed report of the *C. longa* mitochondrial genome, enhancing genomic resources for *Curcuma*. The results improve our understanding of organellar genome diversity and evolution in Zingiberaceae and offer a basis for further comparative studies of plant mitochondrial genomes.

## Figures and Tables

**Table 1:** Gene composition of the *C. longa* mitochondrial genome.

Gene Category	Functional Group	Name of Genes
Core genes	ATP synthase	*atp1*, *atp4* (2), *atp6*, *atp8*, *atp9* (2)
	Cytochrome c biogenesis	*ccmB*, *ccmC*, *ccmFC* *, *ccmFN*
	Ubiquinol cytochrome c reductase	*cob*
	Cytochrome c oxidase	*cox1*, *cox2* **, *cox3*
	Maturases	*matR*
	Transport membrane protein	*mttB*
	NADH dehydrogenase	*nad1* ****, *nad2* ****, *nad3*, *nad4* ***, *nad4L* (2), *nad5* *, *nad6*, *nad7* ***, *nad9*
Variable genes	Ribosomal proteins (LSU)	*rpl2*, *rpl5*, *rpl16*
	Ribosomal proteins (SSU)	*rps1*, *rps2*, *rps3* *, *rps4*, *rps7*, *rps10* *, *rps12*, *rps13*, *rps14*, *rps19*
rRNA	Ribosomal RNAs	*rrn18*, *rrn26*, *rrn5*
tRNA	Transfer RNAs	*trnC-GCA* (2), *trnD-GUC* (3), *trnF-GAA*, *trnE-UUC* (2), *trnG-GCC*, *trnK-UUU* (2), *trnH-GUG* (2), *trnM-CAU* (8), *trnT-UGU*, *trnS-GGA* (2), *trnN-GUU* (3), *trnV-GAC*, *trnH-GUG* (2), *trnS-UGA* (3), *trnW-CCA* (2), *trnS-GCU* (3), *trnY-GUA*, *trnL-CAA*, *trnL-UAG*, *trnR-ACG*, *trnQ-UUG*, *trnP-UGG*

Note: Asterisks (*) indicate the number of introns. *, **, ***, and **** represent one, two, three, and four introns, respectively. Numbers in parentheses indicate the copy number of multi-copy genes.

## Data Availability

The complete mitogenome and plastomes of *C. longa* in this study were submitted to the NCBI database (https://www.ncbi.nlm.nih.gov/, (accessed on 20 January 2026)) under the accession PX870607-PX870618 and PX853824. Genomes and raw genome sequencing data can be found from previously published articles [30].

## Supplementary Materials

The following supporting information can be downloaded at: https://www.mdpi.com/article/10.3390/genes17020243/s1, Figure S1: Circular annotation map of the *C. longa* plastid genome.; Table S1: Genes contained in the plastid genome sequence of *C. longa*; Table S2: Functional genes and organization of the mitogenome of *C. longa*; Table S3: Relative synonymous codon usage for each amino acid pair in the mitochondrial genome of *C. longa*; Table S4: The statistics in ENC of *C. longa*.

## Abbreviations

The following abbreviations are used in this manuscript:

SSR	simple sequence repeat
TR	Tandem repeat
NUMT	Mitochondrion-to-nuclear DNA transfer
NUPT	Plastid-to-nuclear DNA transfer
MTPT	Plastid-to-mitochondrion DNA transfer
NU	Nuclear genome
MT	Mitochondrial genome
PT	Plastid genome
