# Supplementary material for: The Mitochondrial Genome of Curcuma longa: A Large and Structurally Complex Genome with Extensive Intracellular DNA Transfer
Source: Genes (Basel). 2026 Feb 19;17(2):243. doi: 10.3390/genes17020243 (PMC12940290; doi:10.3390/genes17020243)
Supplement: Supplementary file 1 [file genes-17-00243-s001.zip › Table S1.pdf]

Table S1. Genes contained in the plastid genome sequence of *C. longa*.

Gene Category	Functional Group	Name of Genes
Photosynthesis	Subunits of ATP synthase	*atpA, atpB, atpE, atpF *, atpH, atpI*
	Subunits of photosystem I	*psaA, psaB, psaC, psaI, psaJ*
	Subunits of photosystem II	*psbA, psbB, psbC, psbD, psbE, psbF, psbH, psbI, psbJ, psbK, psbL, psbM, psbN, psbT, psbZ*
	Subunits of NADH-dehydrogenase	*ndhA *, ndhB ** (2), *ndhC, ndhD, ndhE, ndhF, ndhG, ndhH, ndhI, ndhJ, ndhK*
	Subunits of cytochrome b/f complex	*petA, petB *, petD *, petG, petL, petN*
	Subunits of rubisco	*rbcL*
Self-replication	Large subunit of ribosome	*rpl2 ** (2), *rpl14, rpl16 *, rpl20, rpl22, rpl23* (2), *rpl33, rpl36*
	DNA dependent RNA polymerase	*rpoA, rpoB, rpoC1 *, rpoC2*
	Small subunit of ribosome	*rps2, rps3, rps4, rps7* (2), *rps8, rps11, rps12 *** (2), *rps14, rps15, rps16 *, rps18, rps19* (2)
	Ribosomal RNAs	*rrn4.5S* (2), *rrn5S* (2), *rrn16S* (2), *rrn23S* (2)
	Transfer RNAs	*trnA-UGC* (2), *trnC-GCA, trnD-GUC, trnE-UUC, trnF-GAA, trnfM-CAU trnG-UCC, trnG-GCC, trnH-GUG* (2); *trnI-CAU* (2), *trnI-GAU* (2), *trnK-UUU, trnL-CAA* (2), *trnL-UAA, trnL-UAG, trnM-CAU, trnN-GUU* (2), *trnP-UGG, trnQ-UUG, trnR-ACG* (2), *trnR-UCU, trnS-GCU, trnS-GGA* (2), *trnS-UGA, trnT-GGU, trnT-UGU, trnV-GAC* (2), *trnV-UAC, trnW-CCA, trnY-GUA*
	Acetyl-CoA carboxylase	*accD*
	c-type cytochrome synthesis gene	*ccsA*
	Envelope membrane protein	*cemA*
	Translational initiation factor	*infA*
	Protease	*clpP ***
	Maturase	*matK*
Unkown	Conserved open reading frames	*ycf1*(2), *ycf2* (2), *ycf3 **, ycf4*

Note: Asterisks (*) indicate the number of introns, * and ** indicate one and two introns, respectively. Numbers in parentheses indicate the copy number of multi-copy genes.
