# Supplementary figures and images for: The Mitochondrial Genome of Curcuma longa: A Large and Structurally Complex Genome with Extensive Intracellular DNA Transfer
Source: Genes (Basel). 2026 Feb 19;17(2):243. doi: 10.3390/genes17020243 (PMC12940290; doi:10.3390/genes17020243)

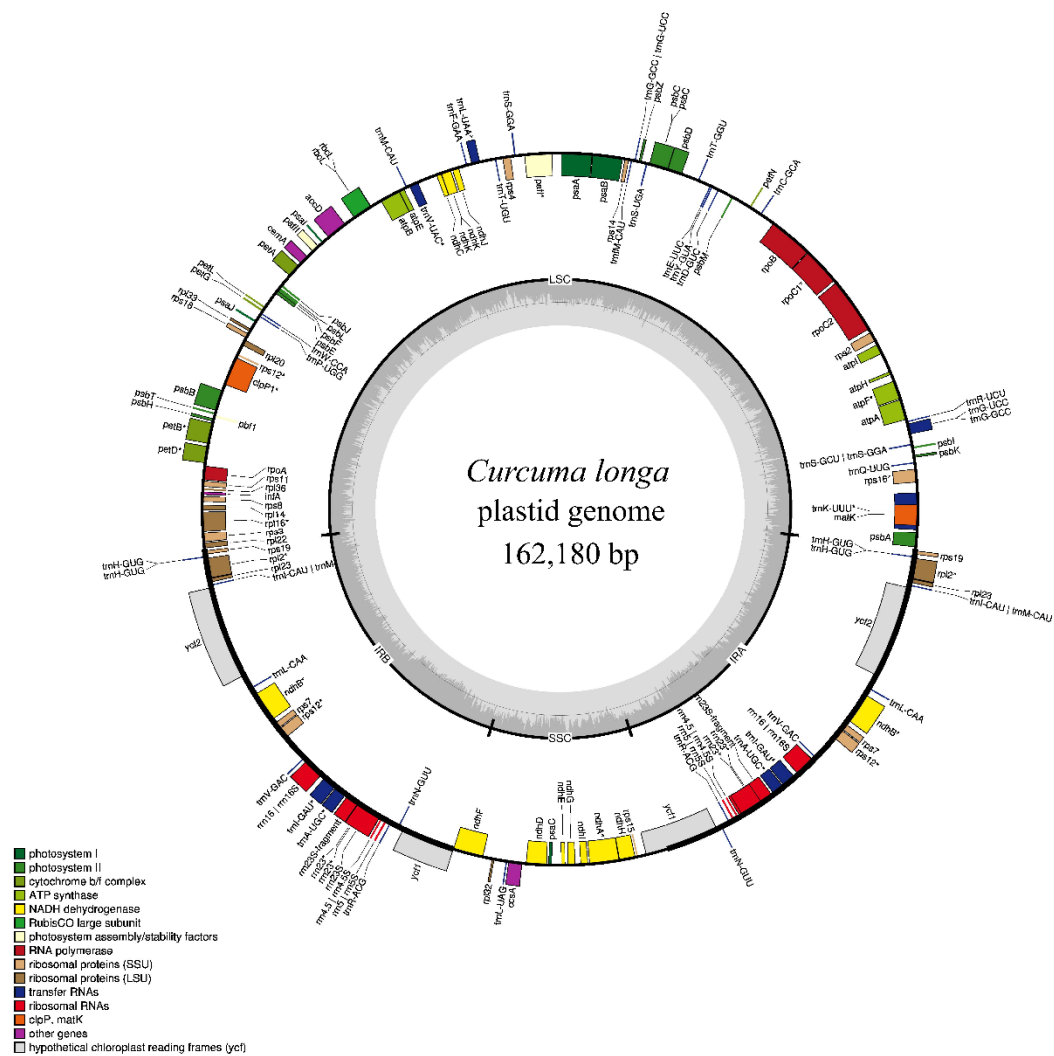

**Figure S1.** Circular annotation map of the *C. longa* plastid genome.

Supplement: Supplementary file 1 [file genes-17-00243-s001.zip › Figure S1.pdf]
